# Relationships Between Depressive Symptoms, Interpersonal Sensitivity and Social Support of Employees Before and During the COVID-19 Epidemic: A Cross-lag Study

**DOI:** 10.3389/fpsyg.2022.742381

**Published:** 2022-03-07

**Authors:** Songli Mei, Cuicui Meng, Yueyang Hu, Xinmeng Guo, Jianping Lv, Zeying Qin, Leilei Liang, Chuanen Li, Junsong Fei, Ruilin Cao, Yuanchao Hu

**Affiliations:** Department of Social Medicine and Health Management, School of Public Health, Jilin University, Changchun, China

**Keywords:** depressive symptoms, interpersonal sensitivity, social support, cross-lag, COVID-19

## Abstract

This study examined the correlation between depressive symptoms, interpersonal sensitivity, and social support before and during the COVID-19 pandemic and verified causal relationships among them. The study used Social Support Scale and Symptom Self-Rating Scale to investigate relevant variables. A total of 1,414 employees from company were recruited for this longitudinal study, which a follow up study was conducted on the same group of participants 1 year later. Paired sample *t*-test results showed that significant differences were only found in social support, not in depressive symptoms or interpersonal sensitivity. The results of correlation analysis showed that social support, depressive symptoms, and interpersonal sensitivity were significantly correlated between wave 1 and wave 2. The cross-lag autoregressive pathway showed that employees’ social support level, depressive symptoms, and interpersonal sensitivity all showed moderate stability. Crossing paths showed that wave 1 social support could significantly predict wave 2 depressive symptoms (*β* = −0.21, *p* < 0.001) and wave 2 interpersonal sensitivity (*β* = −0.21, *p* < 0.001). Wave 1 depressive symptoms (*β* = −0.10, *p* < 0.01) could significantly predict wave 2 social support, while wave 1 interpersonal sensitivity (*β* = 0.07, *p* = 0.10) could not predict wave 2 social support. Social support can be considered as a protective factor against mental health problems.

## Introduction

Since the outbreak of COVID-19 in 2019, 1.9 million confirmed cases and more than 30,000 deaths have been reported globally as of 31 August 2020 (WHO, 2020). In order to prevent the spread of COVID-19, many countries have actively adopted to protective alienation measures, such as social isolation. Changes in lifestyle and limited transportation may cause negative emotions among residents and affect their mental health status (Lemanska et al., 2021). A meta-analysis conducted during the outbreak showed that the global prevalence of depression during COVID-19 was seven times (25.00%) higher than the estimated global prevalence of depression in 2017 (3.44%) (Bueno-Notivol et al., 2021). In some economically developed countries, sick leave due to mental health problems, such as depressive symptom, has increased in recent years (Henderson et al., 2014). Employees suffering from depressive symptom were more likely to commit suicide (Lueck, 2019), violence (Choi et al., 2010), and other risky behaviors that may endanger social order and safety of citizens. On the other hand, the depressive symptoms of employees would lead to the decline of labor productivity and increase the labor production cost (Ammerman et al., 2016). According to the latest research of the World Health Organization, depressive symptom causes about 1 trillion US dollars of losses to the global economy every year (WHO, 2019). Depressive symptom is the most serious mental health problem affecting employees and businesses.

For majority of people, one third of their lifetime was spent in workplaces with their colleagues. According to Mayo’s (Yin and Yin, 2012) theory of interpersonal relationships, workers will have certain informal circles, and such relationships can improve work efficiency and increase the sense of belonging. People with high level of interpersonal sensitivity tend to be more sensitive to other people’s attitudes and opinions toward themselves (Bell and Freeman, 2014). When engage in interpersonal communications with colleagues, this type of people often have a sense of inferiority and discomfort, which could turn into social fear and self-doubt and led to low sociability (Derogatis and Melisaratos, 1983). The outbreak of COVID-19 causes people to have a strong sense of distrust, nervousness, and overreaction to people around them. People from affected areas will feel the panic and unfriendliness of people around them, and have no courage and confidence to socialize, which aggravates the level of interpersonal sensitivity of individuals (Su et al., 2020).

As a resource to protect physical and mental health, social support plays an important role in reducing depressive symptoms and interpersonal sensitivity. Social support is defined as any tool, information, and emotional support provided to an individual by a social network composed of family members, friends, and colleagues (Cohen, 2004). High quality of social support can not only provide protection for individuals during the epidemic, but also maintain good emotional experience networks for individuals (Bergeron et al., 2007). Studies have shown a correlation between depressive symptoms and low quality of family and peer support in employees during the epidemic (Suhail et al., 2021). At the same time, people with more social support and close relationships with family and friends were less likely to report depressive symptoms (Peirce et al., 2000).

As a personality trait, interpersonal sensitivity has been proved to be an unstable characteristic, and it is likely to be affected by any external factors (Mandel et al., 2018). In an investigation of the impact of social support on mental health, it was proposed that high level of family support and peer support could effectively promote the communication between individuals (Jibeen, 2016), enhance individual’s self-esteem and ability to resist stress, and thus weaken individual’s level of interpersonal sensitivity (Hicdurmaz and Oz, 2016). Similarly, the main-effect model emphasizes that social support is an independent predictor of individual mental health, and it can improve the adverse mental health status caused by interpersonal sensitivity even in the period of epidemic isolation (Dubois et al., 1994).

In conclusion, there are many cross-sectional studies on the relationship between social support and depressive symptoms at present, but there is a lack of research on the correlation between social support and interpersonal sensitivity, and few researchers have studied all three variables together. This study examines the causal relationship between depressive symptoms, interpersonal sensitivity, and social support with the longitudinal data gathered on the same sample group before and during the epidemic. Based on literature reviews, we propose two hypotheses. H1: the quality of social support of employees can predict subsequent depressive symptoms and interpersonal sensitivity; H2: Depressive symptoms and interpersonal sensitivity of employees can predict subsequent levels of social support.

## Materials and Methods

### Participants

Cluster random sampling method was used to investigate the employees of a large company in Jilin Province of China. The survey was conducted twice: the first test (wave 1) was conducted in August 2019, and the second measurement (wave 2) was conducted a year later. Employees participated in the study belonged to different sections of the company, including the administrative department, the technical department, the marketing department, the production department, and the logistics department. In the pre-test, 1,650 employees completed the printed questionnaire, of which 51.2% were male and 48.8% were female. In the post-test, after excluded temporary employees, subjects with incomplete information and illogical answers, we used employee’ ID card number to match the data from two times of data collection. In the end, the study obtained 1,414 sets of follow-up data, of which 49.4% were male employees and 50.6% were female employees, losing 236 subjects. The baseline data showed the study included 9.9% employees from the administrative department, 16.6% from the technical department, 36.8% from the marketing department, 29.5% from the production department, and 7.2% employees from the logistics department. Before the investigation, this study was approved by the relevant leaders of the company, orally agreed by the respondents, and approved by the Ethics Committee of School of Public Health, Jilin University.

### Measurement

#### Social Support Scale

Social support was measured using the Social Support Scale compiled by Xiao (1994). The scale contains 10 items, which can be divided into three subcategories: objective support (three items), subjective support (four items), and support utilization (three items). For questions 1–4 and 8–10, a four-point Likert scale was adopted. For question 5, the total score was calculated from five items and each item was calculated from none to full support by 1–4 points, respectively. For question 6.7, if the answer “no sources” was 0 points, and if the answer “the following sources” was several points. The overall score for social support was calculated by adding the items together, and the higher the total score, the higher the level of social support. The Cronbach’s α coefficient measured before and after were 0.91 and 0.92, respectively.

#### Symptom Self-Rating Scale

The mental health status was measured by the Symptom Self-Rating Scale compiled by Derogatis et al. (1976), which included 10 subcategories of somatization, anxiety, depressive symptom, interpersonal sensitivity, obsessive symptoms, hostility, terror, paranoia, psychosis, and sleep. It consisted of 90 items, rated on a five-point Likert scale (1 = never 5 = often). The total score was calculated by adding the score of each item, with higher score reflecting poorer mental health, and a factor score of more than 2 meaning positive. The reliability and validity of this scale were well-demonstrated in the Chinese population (Zhou et al., 2021). Mental health status was measured using depressive symptom and interpersonal sensitivity subscales. The Cronbach’s α coefficient measured before and after the two dimensions were 0.96, 0.97 and 0.94, 0.93, respectively.

### Statistical Analyses

The data was imported into statistical analysis software SPSS 24.0 (IBM). After the data was processed by reverse question, validity test, and latent variable score calculation, descriptive analysis was conducted on the tested variables, and Pearson correlation analysis was used to investigate the internal relationship among the variables. Paired *t*-test was used to detect whether there was significant difference between the two measured data, and independent sample *t*-test was used to analyze gender difference on depressive symptom and interpersonal sensitivity during the epidemic. Structural equation modeling analysis was performed using AMOS 22.0 (IBM) to verify the cross-lag model. *χ*^2^ statistical index and root-mean-square approximation error (RMSEA) were used as absolute fitting measures. Incremental fit index (IFI), Tucker-Lewis index (TLI) and goodness of fit index (GFI) were used as incremental fit indexes. Ratio of *χ*^2^/df < 5, RMSEA < 0.08, IFI, TLI, and GFI values >0.9 indicates that the model fits well.

## Results

### Preliminary Analysis

Table 1 provided the descriptive statistics of participants. Table 2 displayed the means, SDs, and correlation coefficient of the variables. The wave 1 social support was negatively correlated with the depressive symptom and interpersonal sensitivity of wave 1 and wave 2, and significantly positively correlated with the wave 2 social support. Similarly, wave 2 social support was negatively correlated with depressive symptom and interpersonal sensitivity of wave 2 and wave 1. Demographic variables have a correlation relationship with research variables. A paired sample *t*-test of the scores from the first and second measures showed a significant difference in social support (*t* = 6.03, *p* < 0.001), the score of the second measurement was smaller than that of the first measurement. There was no significant difference between the pre and post measures of depression (*t* = −1.13, *p* = 0.26) and interpersonal sensitivity (*t* = −0.10, *p* = 0.93). During the COVID-19 pandemic, the detection rate of depressive symptoms and interpersonal sensitivity level among employees was 28.8% and 27.7%, respectively. Independent sample *t*-test results showed significant differences in depressive symptoms (*t* = 9.85, *p* < 0.001) and interpersonal sensitivity (*t* = 10.15, *p* < 0.001) between males and females during the pandemic, with higher levels of depression and interpersonal sensitivity in males than in females.

**Table 1:** Demographic characteristics of the participants (*N* = 1,414).

Variables	Category	*N* (%)
Gender wave 1, wave 2	Female	698 (49.4)
	Male	716 (50.6)
Department (wave 1)	Administrative department	140 (9.9)
	Technical section	235 (16.6)
	Marketing department	520 (36.8)
	Production department	417 (29.5)
	Logistics department	102 (7.2)
Department (wave 2)	Administrative department	123 (8.7)
	Technical section	242 (17.1)	
	Marketing department	529 (37.4)	
	Production department	409 (28.9)	
	Logistics department	111 (7.9)

**Table 2:** Bivariate correlations, means, and SDs of study variables.

Variables	*M*	*SD*	1	2	3	4	5	6
Social support (wave 1)	41.77	10.49	1					
Social support (wave 2)	40.13	12.35	0.61^**^	1				
Interpersonal sensitivity (wave 1)	15.57	7.92	−0.47^**^	−0.32^**^	1			
Interpersonal sensitivity (wave 2)	15.59	8.17	−0.36^**^	−0.49^**^	0.46^**^	1		
Depressive symptom (wave 1)	23.17	11.87	−0.51^**^	−0.36^**^	0.92^**^	0.47^**^	1	
Depressive symptom (wave 2)	23.53	12.28	−0.39^**^	−0.52^**^	0.45^**^	0.94^**^	0.49^**^	1
Gender wave 1, wave 2			0.24^**^	0.34^**^	−0.22^**^	−0.26^**^	−0.21^**^	−0.25^**^
Department (wave 1)			−0.13^**^	−0.11^**^	0.08^**^	0.10^**^	0.09^**^	0.10^**^
Department (wave 2)			−0.13^**^	−0.12^**^	0.07^**^	0.08^**^	0.07^**^	0.08^**^

Wave 1 represents pre-test, wave 2 represents post-test.

** *p* < 0.01.

### Cross-lag Model

**Figure 1:**
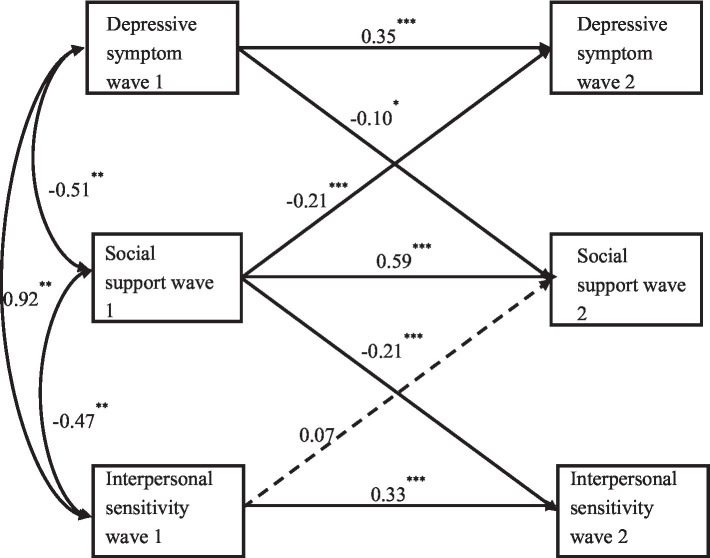
Standardized path coefficient of cross-lag model. One-way arrows with dotted lines represent paths with no significant relationship between two variables. Single arrows with solid lines indicate the path after the crossing; Double arrows indicate the concurrent covariance within the wave 1 variable. The figure does not show the concurrency covariance within the wave 2 variable. Wave 1 = Baseline, wave 2 = 1 year later. ^*^p < 0.05, ^**^p < 0.01, and ^***^p < 0.001.

Figure 1 showed the complete model of cross-lagged paths and autoregressive paths. The model used all the data of the measured variables. The results showed that the model has good fitting indicators (*χ*^2^/df = 4.854, RMSEA = 0.052, IFI = 0.998, TLI = 0.992, and GFI = 0.998). The autoregressive path analysis results of the same variable at different time points showed that the employee’s social support showed high stability, with an autoregressive coefficient of 0.59. Interpersonal sensitivity and depressive symptom showed moderate stability at the two time points, with autoregressive coefficients ranging from 0.33 to 0.35. The results of cross regression path analysis showed that wave 1 social support has significant predictions for wave 2 depressive symptom (*β* = −0.21, *p* < 0.001) and wave 2 interpersonal sensitivity (*β* = −0.21, *p* < 0.001). Wave 1 interpersonal sensitivity (*β* = 0.07, *p* = 0.10) did not significantly predict wave 2 social support, but wave 1 depressive symptom (*β* = −0.10, *p* < 0.01) has a significant predictive effect on the wave 2 social support.

## Discussion

The study used two sets of data from a longitudinal study conducted in Jilin Province of China to explore the relationship between depressive symptoms, interpersonal sensitivity, and social support. Paired sample *t*-test was used to test whether there were differences between two sets of data, independent sample *t*-test was used to analyze gender differences on depressive symptom and interpersonal sensitivity during the epidemic, and cross-lag model was used to verify the mutual predictive effect among depressive symptoms, interpersonal sensitivity, and social support. The study found that there were significant differences in the level of social support at both times point of measurement. However, the level of social support was lower during the epidemic compare to the time when epidemic did not occur, which was consistent with previous researches (Savolainen et al., 2021). COVID-19 was a virus with high infectious rate and severe health consequences, which forced countries to deploy extreme measures to contain the spread. When an employee needed help or comfort, his colleagues or friends, considering the current situation of the epidemic, can only offer support and encouragement online. Studies have shown that face-to-face communication and physical contact make people feel better than online greetings (Macias et al., 2013). As a result, individuals’ perceived levels of social support were lower than they were before the pandemic. The research results showed that the detection rate of depression and interpersonal sensitivity during the epidemic was lower than other studies (Su et al., 2020). By the time of second data collection, the pandemic situation had been effectively controlled. The public has gained confidence in the government’s rapid and effective prevention and control measures, they also gained a better understanding of health information about novel coronavirus pneumonia, and reduce the panic caused by misinterpretations. The state’s strong control strategies and individual’s correct perceptions reduced the psychological distress and improved the mental health of individuals.

There was no significant difference between depressive symptom and interpersonal sensitivity by paired sample *t*-test, and it was different from previous studies (Gallagher and Wetherell, 2020; Jiang, 2020). Possible reason was as follows, as of 31 August 2020, 138 cases of COVID-19 patient have been reported in Jilin Province, among which 136 cases have been cured and discharged (People, 2020). Since the outbreak of the new coronavirus in Jilin Province, good protective measures have been taken. The number of people infected by the new coronavirus was relatively small, and the stimulus-response theory (Jacoby, 2002) in psychology showed that the external environmental stimulus can significantly affect people’s psychological behavior. The situation of COVID-19 infection in Jilin Province is far less than that in other provinces. This kind of stimulus in quantity will reduce public fear about the epidemic, and make people reclaim their calmness and have positive state of minds. Independent sample *t*-test results show significant gender differences in depressive symptoms and interpersonal sensitivity during the COVID-19 pandemic. More severe depressive symptoms and higher interpersonal sensitivity in men than women, which is different compared to other study results (Vloo et al., 2021). According to Chinese traditional gender roles and division of labor, men bear more economic responsibilities in the family, but the economic downturn caused by the epidemic and the implementation of layoff announced by many companies greatly increased the psychological pressure in men (Ren et al., 2020). Compared to women, men have more active and frequent social activities (Olaseni et al., 2020), but preventive measures like social isolation and family isolation limited these activities, causing men to feel more socially isolated, and negatively affected their mental health status. Studies have shown that men tend to reduce stress by addressing problems caused by stressors, while women turn to psychological adaptation (Liu et al., 2021). Under stay-at-home orders and social distance policy, men worried about their status of employment and social relationships, but have no solution to resolve the problem which caused more psychological distress.

It is found that wave 1 social support can predict wave 2 depressive symptom and wave 2 interpersonal sensitivity, the results validate hypothesis 1. This indicates that higher level of social support can reduce severity of depressive symptoms and interpersonal sensitivity of individuals, which also confirms the protective effects of social support on individual’s mental health status (Schug et al., 2021). As an important environmental resource (Thoits, 2011a), social support affects people’s physical and mental health and behavioral patterns, and can effectively get help from their own support system, which is closely related to the control and prevention of depressive symptoms (Thoits, 2011b). During the COVID-19 pandemic, employees were exposed to multiple stressors (for example, the pressure of layoffs, the pressure of fear of infection), which increases the likelihood of individuals suffering from depression (Knolle et al., 2021). Social support can make individuals who were under pressure more easily obtain self-esteem and self-efficacy, enhance their coping ability and reduce the harm caused by stress, and resist the occurrence of negative emotions such as depressive symptom (Lee et al., 2014). A large number of studies have proved that social support has a buffer effect on pressure (Cohen and Wills, 1985; Yu et al., 2021). When employees suffered depressive symptoms caused by multiple pressures, understanding from family members, help from colleagues and friends can make employees feel warm and full of hope and expectation for life and the future. The existence of social support can effectively reduce the intensity of the relationship between stressful events and depressive symptoms, so as to prevent or reduce the possibility of depressive symptom.

A survey on employees’ social support and interpersonal helping behaviors showed that mutual help among employees can reduce individuals’ rejection of colleagues’ interpersonal interference, enhance the trust and communication depth between them, and thus reduce the severity of interpersonal sensitivity (Horita and Otsuka, 2014). When employees have difficulties in interpersonal communication, support and tolerance from family and peers can help employees with sensitive interpersonal relationship to find confidence in interpersonal communication, be willing and take the initiative to conduct interpersonal communication, and then change the personality traits of sensitive interpersonal relationship. And good interpersonal relationship can make it easier for individuals to stimulate intrinsic motivation when facing setbacks and pressures, and seek effective ways to deal with challenges, thus effectively preventing the possible mental health problems of individuals.

The study demonstrated that wave 1 depressive symptom significantly predicted wave 2 social support, consistent with previous research (Tao and Li, 2003) and this result confirmed part of Hypothesis 2 employees with depressive symptoms will demonstrate low mood, dull thinking and reduced volitional activity. Consequentially, these individuals will have low self-evaluation, which can create the sense of uselessness and worthlessness in them. Employees begin to become careless about everything around them, avoid and refuse social communications and interactions. Research of Nakayama and Amagasa (2004) found that when an employee looks depressed, some people try to cheer him up, while others simply leave him alone and let him heal himself. Individuals’ persistent depressive symptoms can erode the empathy and patience of those around them, reducing the social support that employees can receive. In addition, Beck’s cognitive model of depression pointed out that depressed individuals have cognitive biases, tend to ignore positive information and pay more attention to negative information, and negatively coded and interpreted events (Monsalve et al., 2021). Due to cognitive bias, depressed individuals interpret the help offered by family members or colleagues negatively and give relatively bad responses, which is manifested as the predictive negatively effect of employee depressive symptom on social support. Unlike previous studies (Lin, 2017), this study found that interpersonal sensitivity level from wave 1 did not significantly predict the low level of social support in wave 2. This outcome could be explained by the COVID-19 outbreak in 2019. The pneumonia epidemic forced employees to respond to the national policies like stay-at-home orders, which greatly limited the social contact and communication between People and their colleagues and friends. Even after return to work in 2020, companies took prevention measures, like allowed their employees to work from home and limited group activities at workplace etc. These measures limited the interaction and communication between employees. Interpersonal sensitivity is a type of personality trait, it is a psychological movement which can only be observed as a behavior pattern. When people have less opportunity to interact with each other, they will have less opportunity to observe other’s behaviors, and the problem of strong interpersonal sensitivity cannot be shown. Thus, the influence of interpersonal sensitivity on the level of social support was limited.

## Limitations

There were some limitations in this study. First, the depressive symptom and interpersonal sensitivity variables being investigated in this study were all came from the same source, the self-rating symptom scale. Therefore, there may be some deviation in the measurement of the real situation of the surveyor. Moreover, this study only studied the relationships between depressive symptom, interpersonal sensitivity, and social support variables, and lacked the investigation research on the internal influencing factors. Finally, data were collected from self-reported questionnaires, which may have social desirability bias.

## Conclusion

This study provided longitudinal evidence of temporal interrelationship between depressive symptom, interpersonal sensitivity, and social support. The cross-lag model showed a dynamic relationship between the social supports that employees can receive and their mental health status over time. Social support was a reliable predictor of future individual depressive symptoms and interpersonal sensitivity. Depressive symptom was an important predictor for social support, whereas, interpersonal sensitivity was considered not a predictor for social support. The results of this study can help clarify the different mechanisms among depressive symptom, interpersonal sensitivity, and social support, and help employees to realize the importance of social support in improving mental health.

## Data Availability Statement

The datasets presented in this article are not readily available because only this research group can carry out related research. Requests to access the datasets should be directed to YaH, hu_yuanchao@sohu.com.

## Ethics Statement

The studies involving human participants were reviewed and approved by the Ethics Committee of School of Public Health, Jilin University. The patients/participants provided their written informed consent to participate in this study.

## Author Contributions

SM: conceptualization, data curation, formal analysis, validation, writing—original draft, and writing—review and editing. CM and YeH conceptualization, data curation, and writing—review and editing. XG: conceptualization and writing—review and editing. JL, ZQ, LL, CL, RC, and JF: writing—review and editing. YaH conceptualization, data curation, funding acquisition, supervision, and writing—review and editing. All authors contributed to the article and approved the submitted version.

## Funding

This study was supported from research on innovation and development strategy of Science and Technology Department of Jilin Province (20210601140FG).

## Conflict of Interest

The authors declare that the research was conducted in the absence of any commercial or financial relationships that could be construed as a potential conflict of interest.

## Publisher’s Note

All claims expressed in this article are solely those of the authors and do not necessarily represent those of their affiliated organizations, or those of the publisher, the editors and the reviewers. Any product that may be evaluated in this article, or claim that may be made by its manufacturer, is not guaranteed or endorsed by the publisher.
